# Comparative Evaluation of Infrared Thermography and Mammography in the Detection of Breast Cancer

**DOI:** 10.7759/cureus.101203

**Published:** 2026-01-09

**Authors:** Harish S, Sudha K Das, Sapna Patel MC, Deepak Naik, Sachin K, Ganashree M H, Sharon Esther, Sankar Chandra Vadan Dhanekula

**Affiliations:** 1 Department of General Surgery, Jagadguru Sri Shivarathreeshwara (JSS) Medical College and Hospital, Mysore, IND; 2 Department of Radiology, Jagadguru Sri Shivarathreeshwara (JSS) Medical College and Hospital, Mysore, IND; 3 Department of Pathology, Jagadguru Sri Shivarathreeshwara (JSS) Medical College and Hospital, Mysore, IND

**Keywords:** artificial intelligence, breast cancer, early detection, infrared thermography, low-resource settings, mammography, thermal imaging

## Abstract

Objective: The primary objective of this study was to evaluate the diagnostic accuracy of infrared thermography for detecting breast carcinoma and to compare its performance with that of standard mammography. A secondary objective was to assess the potential role of thermography as a supplementary, non-invasive, radiation-free imaging modality, particularly in settings with limited access to conventional breast imaging.

Methodology: A diagnostic comparison study was conducted over 18 months at JSS Hospitals, Mysuru, involving 30 female patients aged 20-60 years with breast tumors. All participants underwent infrared thermography, mammography, and histopathological analysis. Thermograms showing temperature variations of ≥3°C were considered malignant. Data were analyzed using IBM SPSS Statistics for Windows, Version 28 (Released 2021; IBM Corp., Armonk, New York), and diagnostic accuracy was assessed based on sensitivity, specificity, positive predictive value (PPV), and negative predictive value (NPV), with statistical significance set at p < 0.05.

Results: Of the 34 breast lesions evaluated, 24 (70.6%) were malignant and 10 (29.4%) were benign. On mammography, positive findings were observed in 23 (95.8%) malignant cases and one (10%) benign case. Infrared thermography demonstrated no thermal change in all benign and six (25%) malignant cases. Mammography detected 23 of 24 malignant lesions, yielding an area under the curve (AUC) of 0.967 (p < 0.05). Infrared thermography detected 18 of 24 malignant lesions, with an AUC of 0.875 (p < 0.001).

Conclusion: Infrared thermography demonstrated high specificity and a strong PPV; however, its low sensitivity limits its standalone diagnostic utility in breast cancer detection. Nevertheless, owing to its cost-effectiveness, portability, and radiation-free properties, thermography may serve as a valuable supplementary tool, particularly in low-resource settings and among younger women with dense breast tissue.

## Introduction

Breast cancer is the most frequently diagnosed malignancy in women and remains the leading cause of cancer-related mortality worldwide [1]. Between the 1980s and 2020, high-income countries experienced a 40% reduction in mortality, attributed to improved access to cancer detection and treatment [2]. Despite significant advances, breast cancer continues to be a primary global health concern, with an estimated 2.3 million new cases and 670,000 deaths among women worldwide in 2022. In India, it accounts for 13.7% of all cancer cases and 10.7% of cancer-related mortality [3]. Globally, the incidence of breast cancer among younger women is rising [4,5]. In Asia, including India, approximately 50% of cases are diagnosed at stages III and IV, compared with only 12% in the United States [6].

In Asian and African countries, the cost of breast cancer treatment is approximately USD 390 per disability-adjusted life year (DALY) averted when diagnosed at stages I-III but increases to about USD 3,900 per DALY when diagnosed at stage IV. Delayed diagnosis is associated with substantially higher mortality and morbidity [7].

Timely detection is essential for reducing mortality and improving survival outcomes. While self-breast examination (SBE) is occasionally practiced, its sensitivity for cancer detection is limited. Mammography remains the principal FDA-endorsed screening tool and is widely used in high-income countries [8]. However, its effectiveness decreases in women with dense breast tissue, and it is associated with higher false-positive rates [9]. Additionally, mammography can cause temporary discomfort and involves exposure to low-dose ionizing radiation; however, according to major radiological and oncological societies such as the American College of Radiology (ACR) [10] and the International Commission on Radiological Protection (ICRP) [11], this exposure carries a minimal cancer risk that is far outweighed by the substantial benefits of early breast cancer detection. Other imaging modalities, such as magnetic resonance imaging (MRI) and ultrasound, are often used in conjunction with mammography. MRI provides higher sensitivity but is costly [12]. In contrast, ultrasound plays a complementary role, not only differentiating between cystic and solid lesions but also contributing to lesion characterization, biopsy guidance, axillary assessment, and evaluation of vascularity and tissue stiffness using Doppler and elastography techniques. Furthermore, breast ultrasound is particularly valuable in screening women with dense breasts, monitoring response to neoadjuvant therapy, and assisting in preoperative planning, as emphasized in the ACR Breast Imaging Reporting and Data System (BI-RADS) Atlas and recent European Society of Breast Imaging (EUSOBI) recommendations [13,14].

Compared with mammography, thermography is a non-invasive, non-ionizing, and more cost-effective imaging technique. It replaces expensive X-ray-based equipment with a relatively economical infrared camera. Thermography detects infrared radiation emitted from the skin surface, which reflects underlying subcutaneous heat and vascular activity [15]. Malignant tumors exhibit increased metabolic activity that raises the temperature of surrounding tissues through angiogenesis and vasodilation, thereby increasing local blood flow [9]. Infrared cameras can detect these temperature changes caused by heightened metabolism and circulation [9]. However, elevated breast temperature is not specific to malignancy and may also result from bacterial or viral infections or other inflammatory conditions, which can complicate diagnostic interpretation [10]. Despite this limitation, thermography is more cost-effective than mammography and has demonstrated better performance than SBE and clinical breast examination (CBE), making it a potentially valuable screening tool in resource-limited and developing settings [15].

Unlike mammography, thermography has the potential to detect early physiological changes associated with breast cancer, potentially allowing identification at an earlier stage of disease development [9]. In addition, thermography does not involve ionizing radiation, making it suitable for women of all ages from preadolescence to postmenopause, including those with dense or fibrocystic breast tissue, as well as pregnant or lactating women [15].

In India, barriers to breast cancer screening include a large rural population, the high cost and limited availability of mammography, and a shortage of trained healthcare personnel. These challenges reduce the effectiveness of current screening programs [16]. Therefore, there is an urgent need for a diagnostic approach that is both affordable and accessible. The primary objective of this study was to evaluate the diagnostic accuracy of infrared thermography for detecting breast carcinoma and to compare its performance with that of standard mammography. A secondary objective was to assess the potential role of thermography as a supplementary, non-invasive, and radiation-free imaging modality, particularly in settings where access to conventional breast imaging is limited.

## Materials and methods

Study design and settings

This diagnostic comparative study was conducted in the Departments of General Surgery, Surgical Oncology, and Medical Oncology at Jagadguru Sri Shivarathreeshwara (JSS) Hospitals, Mysuru, between June 2023 and December 2025. Over the 18-month study period, the efficacy of infrared thermography for breast cancer detection was evaluated. Eligible patients admitted to the participating departments were enrolled after providing informed consent in their preferred language (English, Kannada, or Hindi).

Sampling technique

A purposive sampling technique was used, assuming a breast cancer prevalence of 2%. The sample size (S) was determined using the standard statistical formula: $S = \frac{Z^{2} \times P \times Q}{D^{2}}$, where Z = 1.96 (for a 95% confidence level), Q = 1 - P = 0.98, and D = 0.05 (allowable margin of error). Substituting these values, the calculated sample size was 64 patients. These 64 eligible patients were subsequently enrolled in the study.

Inclusion and exclusion criteria

Inclusion criteria were female patients aged 20-60 years who presented with breast lumps, were admitted to the hospital, and provided informed consent. Exclusion criteria included male patients, females outside the 20-60 year age range, those with clinically apparent inflammatory breast conditions, and patients receiving chemotherapy or radiotherapy for the same pathology.

Data collection

**Figure 1:**
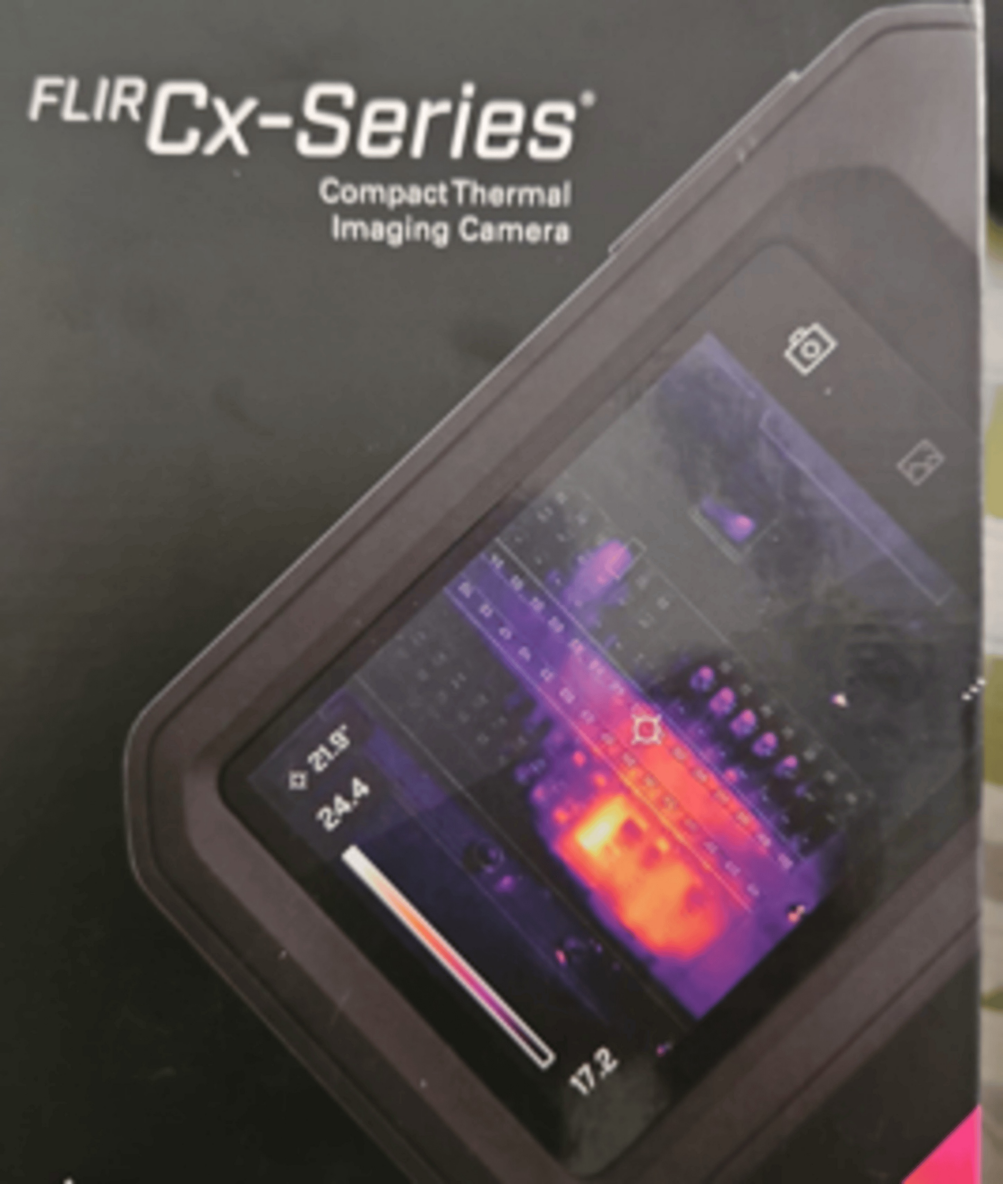
FLIR Cx-Series compact thermal imaging camera displaying a thermal view of an electrical panel.

A focused clinical history was obtained, documenting the duration of the breast lump and any prior investigations, including fine-needle aspiration cytology, mammography, or core needle biopsy. Demographic and clinical parameters such as age, sex, and inpatient number were recorded. Patients were examined in a dimly lit room maintained at 25°C, where, after five minutes of breast exposure, thermographic imaging was performed. Three images were captured from frontal, superior, and lateral views using a FLIR C3 (Teledyne FLIR, Wilsonville, Oregon) compact thermal imaging camera, with emissivity set to 0.98 and the camera positioned approximately 1 m from the patient. The device was calibrated according to the manufacturer’s instructions before image acquisition (Figure 1).

**Figure 2:**
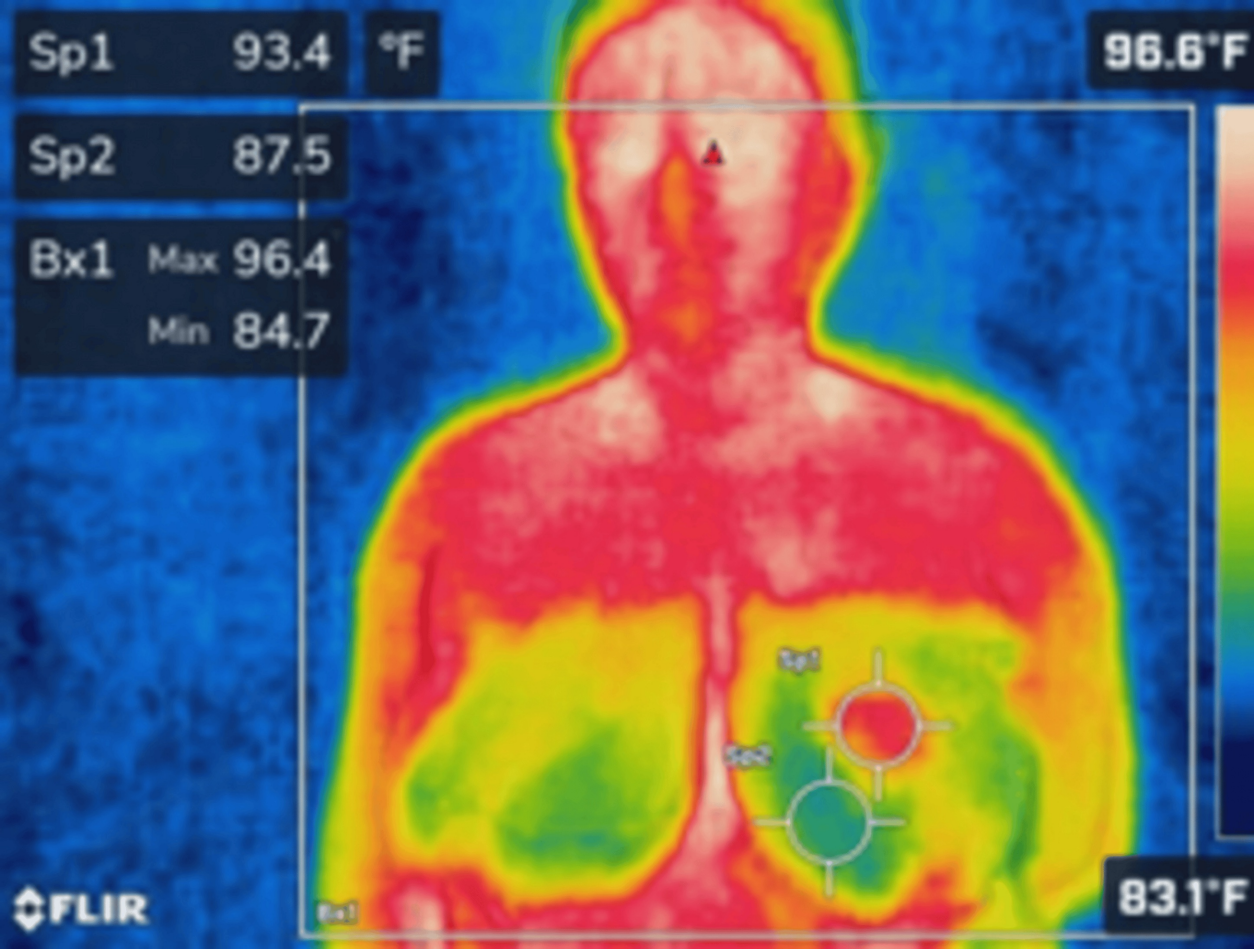
Thermal imaging of the anterior torso using a FLIR camera showing temperature variations across the chest and upper abdomen. The maximum temperature recorded within the region of interest (Bx1) is 96.4°F, with localized measurements at Sp1 (93.4°F) and Sp2 (87.5°F).

**Figure 3:**
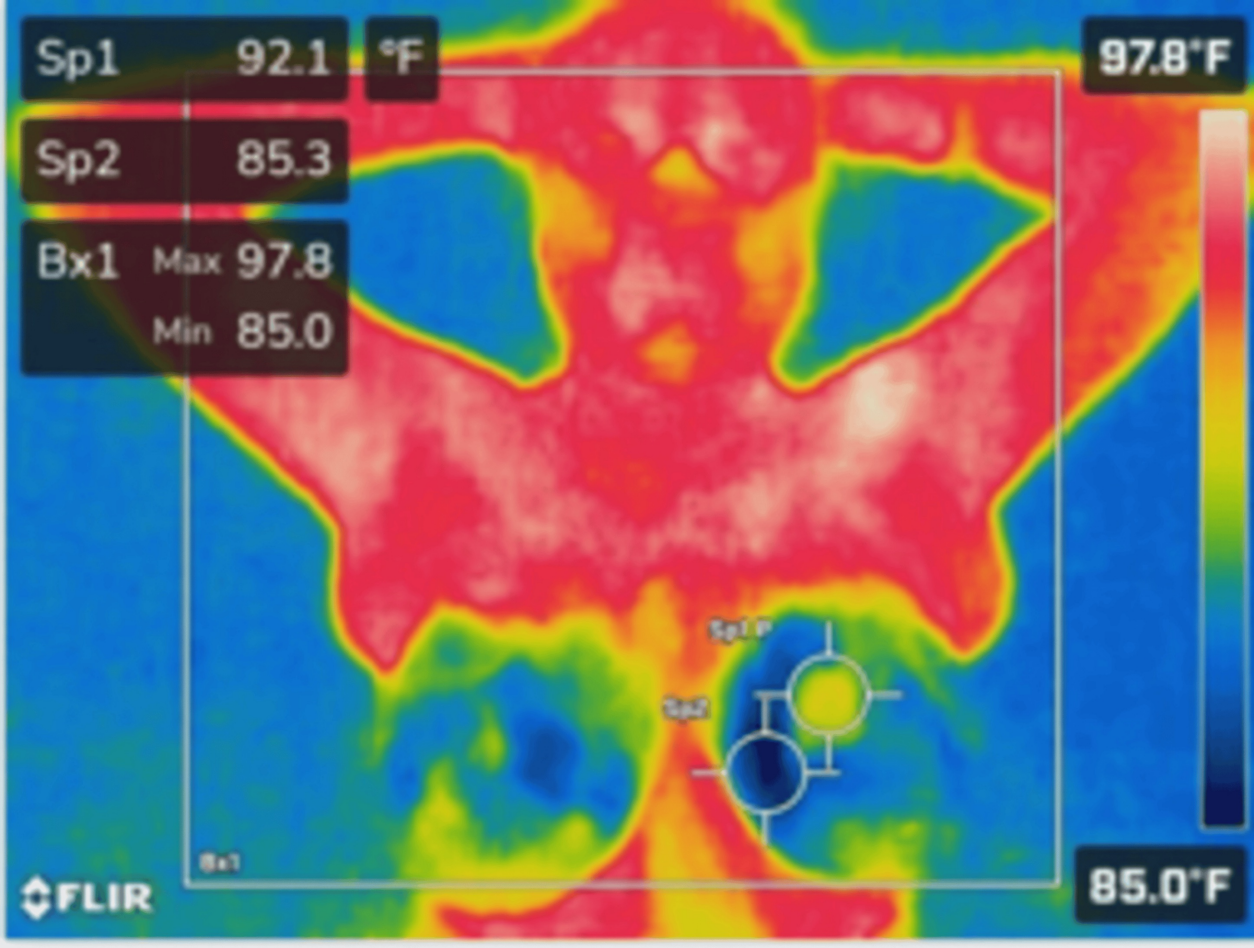
Thermal imaging of the anterior torso with arms raised, highlighting temperature distribution in the lower abdomen and chest area. The highest temperature within the region of interest (Bx1) is 97.8°F, with specific spot readings of Sp1 (92.1°F) and Sp2 (85.3°F).

Thermal images were analyzed using FLIR Tools software by defining a standardized region of interest over the breast tissue, from which maximum and mean temperatures were recorded. Temperature asymmetry across corresponding breast regions was assessed as a qualitative and quantitative parameter. Differences of 2.5-3.0°C were considered suggestive of benign pathology, while higher temperature differentials were considered suspicious for malignancy based on reported physiological patterns of increased vascularity and metabolic activity in malignant lesions. These findings were interpreted in conjunction with mammography results, and histopathology served as the definitive diagnostic standard. Image interpretation was performed independently by two observers who were blinded to mammography and histopathology findings, and inter-observer agreement was assessed using Cohen’s kappa coefficient (Figures 2, 3).

All patients subsequently underwent mammography as part of standard clinical care, with images interpreted independently by two radiologists blinded to thermographic results and lesions classified according to BI-RADS categories. Lesions categorized as BI-RADS 4 or 5 were considered malignant for study purposes, and any discrepancies were resolved by consensus. Following imaging, patients underwent appropriate diagnostic or therapeutic procedures, including fine-needle aspiration cytology, core needle biopsy, lumpectomy, or modified radical mastectomy. Histopathological examination of excised tissue served as the gold standard for validating the findings of infrared thermography and mammography.

Data analysis

Data were summarized using descriptive statistics, including frequencies, percentages, means, and standard deviations (SDs). Categorical variables were compared using the chi-square test. At the same time, diagnostic performance was evaluated by calculating sensitivity, specificity, positive predictive value (PPV), negative predictive value (NPV), and area under the receiver operating characteristic curve (AUC). A p-value ≤ 0.05 was considered statistically significant. All statistical analyses were performed using IBM SPSS Statistics for Windows, Version 28 (Released 2021; IBM Corp., Armonk, New York).

## Results

Among the 34 cases, 10 (29.4%) were benign and 24 (70.6%) were malignant. All benign cases (10, 100%) underwent excision. Malignant cases were managed with lumpectomy in 13 (54.2%) patients and modified radical mastectomy (MRM) in 11 (45.8%) (p < 0.001). Breast density demonstrated a significant association with malignancy (p < 0.001). Pathological analysis revealed type A in nine (90%) of benign cases, whereas malignant cases predominantly exhibited type B in 13 (54.2%) and type C in 9 (37.5%). Left-sided lesions were observed more frequently among malignant cases (13, 54.2%), whereas right-sided lesions predominated in benign cases (7, 70.0%). However, this difference did not reach statistical significance (p = 0.20) and should therefore be interpreted with caution (Table 1).

**Table 1:** Comparison of clinical and radiological parameters between the benign and malignant groups. * represents a significant p-value. A p-value less than 0.05 was considered significant. Data have been presented as N (%) and mean ± SD. MRM: modified radical mastectomy

Parameters	Benign N = 10	Malignant N = 24	Overall N = 34	Student's t-test/chi-square	p-value
Mean age	46.79 ± 11.60	35.3 ± 13.06	51.8 ± 7.07	2.22	0.034*
Type of surgery				34	<0.001*
MRM	0 (0%)	11 (45.83%)	11 (32.25%)		
Lumpectomy	0 (0%)	13 (54.16%)	13 (38.23%)		
Excision	10 (100%)	0 (0%)	10 (29.41%)		
Breast density				23.96	<0.001*
A	9 (90%)	2 (8.33%)	11 (32.35%)		
B	1 (10%)	13 (54.17%)	14 (41.18%)		
C	0 (0%)	9 (37.5%)	9 (26.47%)		
Laterality of lesion				1.61	0.20
Left	3 (30%)	13 (54.17%)	16 (47.06%)		
Right	7 (70%)	11 (45.83%)	18 (52.94%)		

Among the 24 malignant breast lesions, the upper outer quadrant was the most common site, observed in 16 (66.6%) cases. Invasive carcinoma was present in 22 (91.6%) cases, while carcinoma in situ was noted in two (8.3%) cases. High-grade tumors (G3) predominated in 14 (58.3%) cases. Most tumors were stage T2, observed in 16 (66.6%) cases, and Mx was reported in 18 (75%) cases. Axillary lymphadenopathy assessment was not applicable in 10 (41.6%) cases; among the remaining cases, nodal involvement was present in 10 (41.6%) (Table 2).

**Table 2:** Tumor characteristics in malignant breast lesions (N = 24). Data have been presented as N (%).

Parameters	Case Number N = 24
Site of the lesion	
Upper outer quadrant	16 (66.6%)
Upper inner quadrant	8 (33.3%)
Lower outer quadrant	6 (25%)
Lower inner quadrant	3 (12.5%)
Central	1 (4.16%)
Histology type	
Invasive carcinoma	22 (91.6%)
Carcinoma in situ	2 (8.3%)
Grade of the tumor	
G1	3 (12.5%)
G2	8 (33.33%)
G3	14 (58.33%)
M Stage of the tumor	
Mx	18 (75%)
M0	6 (25%)
T Stage of the Tumor	
T1	4 (16.6%)
T2	16 (66.6%)
T3	4 (16.6%)
T4	0 (0%)
Presence of axillary lymphadenopathy	
Not applicable	10 (41.6%)
N0	14 (58.3%)
N1	8 (33.3%)
N2A	1 (4.17%)
N3A	1 (4.17%)

On mammography, positive findings were observed in 23 (95.8%) malignant cases and one (10%) benign case. Infrared thermography demonstrated no thermal change in all benign and six (25%) malignant cases. Among malignant lesions, a temperature difference of 2.0-2.9°C was the most frequent finding, observed in 12 (50%) cases, followed by disagreements of ≥3.0°C in four (16.6%) cases (Table 3).

**Table 3:** Diagnostic outcomes of mammography and infrared thermography in benign and malignant cases. Data have been presented as N (%).

Outcomes	Benign N (%)	Malignant N (%)	Total N (%)
Mammogram findings			
Positive	1 (10%)	23 (95.83%)	24 (92.31%)
Negative	1 (10%)	1 (4.16%)	2 (7.69%)
Infrared thermography			
No change	10 (100%)	6 (25%)	16 (47.06%)
Less than 2D	0 (0%)	2 (8.33%)	2 (5.88%)
2D to 2.9D	0 (0%)	12 (50%)	12 (35.29%)
3D or more	0 (0%)	4 (16.6%)	4 (11.76%)

Mammography demonstrated a sensitivity of 95.8% (95% CI: 78.9-99.9%) and a PPV of 95.8% (95% CI: 85.2-98.9%), with an overall diagnostic accuracy of 92.3% (95% CI: 74.9-99.1%). In comparison, infrared thermography showed a notably lower sensitivity of 75.0% (95% CI: 53.3-90.2%), indicating a reduced ability to detect all malignant cases. Although thermography achieved a specificity of 100% (95% CI: 69.2-100.0%) and a PPV of 100% (95% CI: 81.5-100.0%), its lower sensitivity underscores a significant limitation when compared with mammography. The overall diagnostic accuracy of thermography was 82.4% (95% CI: 65.5-93.2%), suggesting that while thermography may have value as an adjunctive tool, it cannot replace mammography as a primary diagnostic modality (Table 4).

**Table 4:** Diagnostic performance of mammography and infrared thermography.

Parameters	Mammogram		Infrared Thermography	
	Value	95% Confidence Interval	Value	95% Confidence Interval
Sensitivity	95.83%	78.88% to 99.89%	75%	53.29% to 90.23%
Specificity	50%	1.26% to 98.74%	100%	69.15% to 100%
Positive likelihood ratio	1.92	0.48 to 7.68	-	-
Disease prevalence	92.31%	74.87% to 99.05%	70.59%	52.52% to 84.9%
Positive predictive value	95.83%	85.16% to 98.93%	100%	81.47% to 100%
Negative predictive value	50%	8.57% to 91.43%	62.50%	45.46% to 76.92%
Accuracy	92.31%	74.87% to 99.05%	82.35%	65.47% to 93.24%

**Figure 4:**
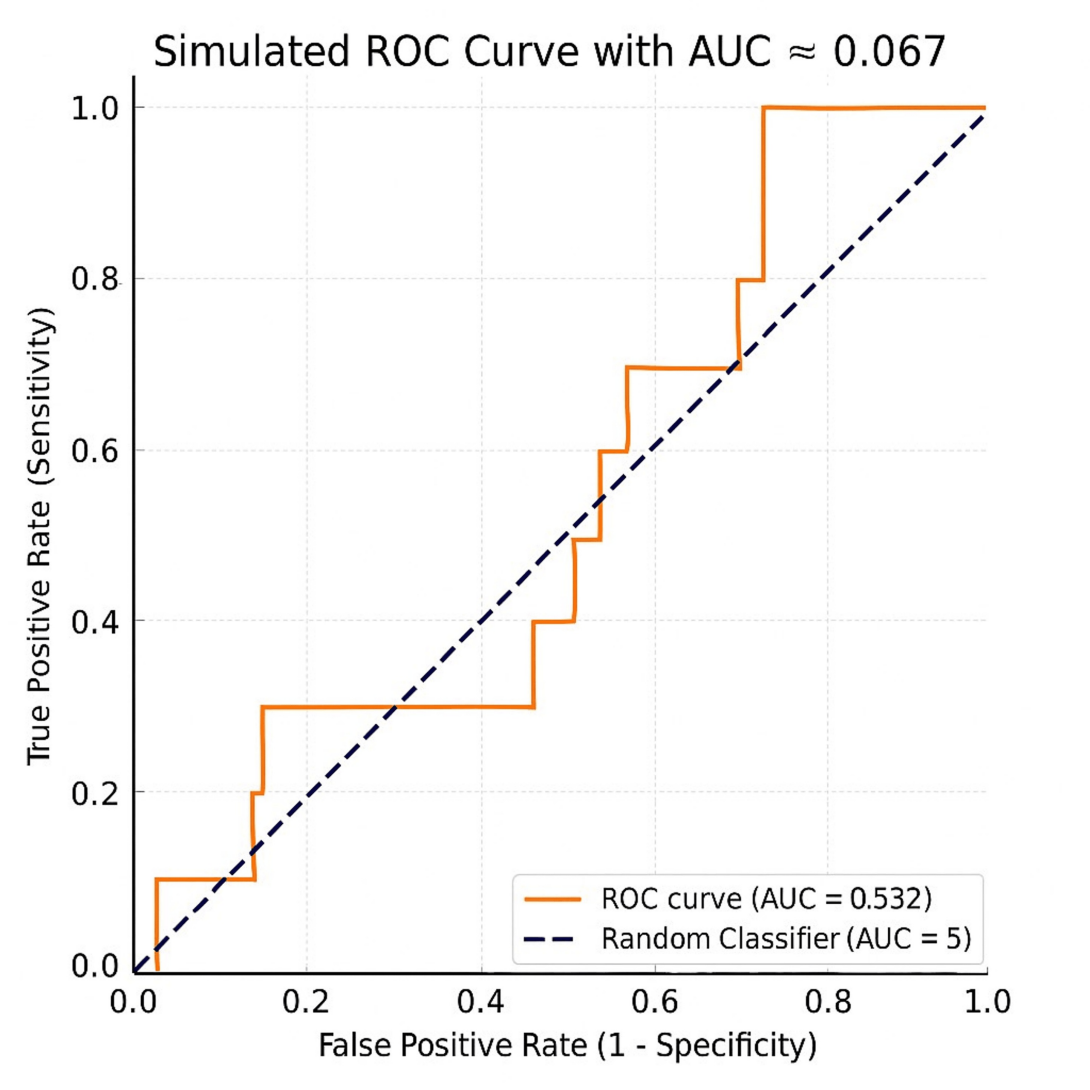
Mammogram findings. Receiver operating characteristic (ROC) curve analysis was performed to evaluate the discriminatory ability of the studied parameter. The area under the ROC curve (AUC) was 0.532, indicating poor diagnostic performance. The ROC curve lay close to the line of no discrimination, suggesting that the parameter had only a marginal ability to differentiate between the two groups, performing only slightly better than random chance (AUC = 0.5). Overall, the findings indicate that the studied variable does not demonstrate sufficient accuracy to be used as a reliable standalone diagnostic or predictive marker. ROC: receiver operating characteristic; AUC: area under the curve

The reliability of mammography in evaluating breast lesions was demonstrated by its overall diagnostic accuracy of 92.3%. Diagnostic performance was further supported by an area under the ROC curve of 0.967, which was statistically significant (p < 0.05). These findings highlight the superior sensitivity and accuracy of mammography compared with infrared thermography, confirming its role as the primary imaging modality for breast cancer detection (Figure 4).

**Figure 5:**
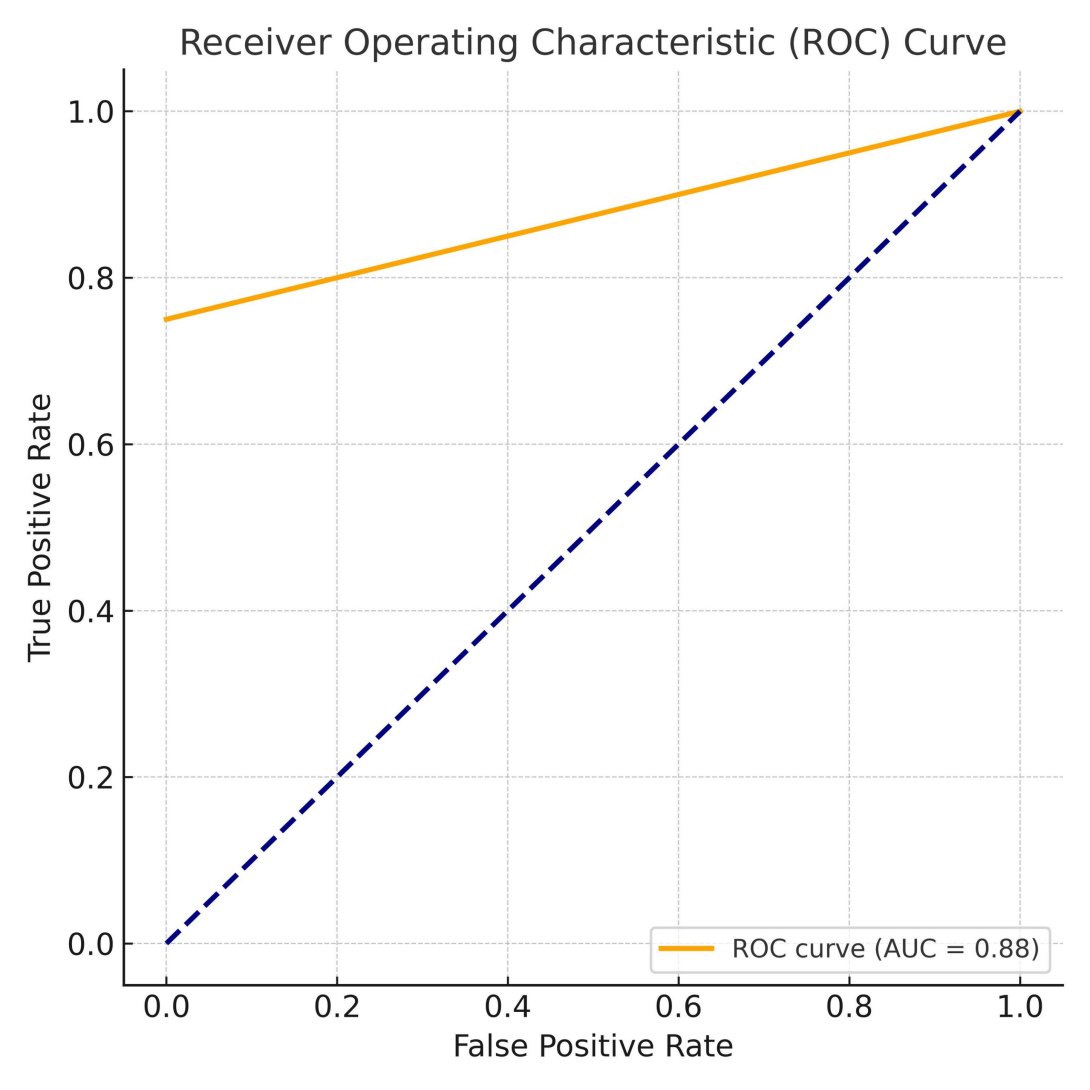
Receiver operating curve for infrared thermography findings. AUC: area under the curve

In Figure 5, the AUC of 0.875 demonstrated strong diagnostic performance, while the p-value of <0.001 confirmed statistical significance. These findings highlight infrared thermography as a valuable adjunct in breast cancer diagnosis, particularly due to its high specificity and excellent PPV. However, its relatively lower sensitivity and NPV suggest that it should be combined with other diagnostic modalities.

## Discussion

The study population had a mean age of 46.79 years (range: 20-60). Subgroup analysis showed that the mean age of patients with malignant lesions was 51.8 ± 7.07 years, whereas that of patients with benign lesions was 35.3 ± 13.06 years. This difference was statistically significant (p = 0.034), indicating a higher prevalence of malignancy among older individuals. These findings are consistent with previous literature emphasizing age as a key risk factor in the development of breast cancer [17,18]. For comparison, Alikhassi et al. [16] reported a mean age of 41.0 ± 10.4 years; Prasad et al. [17] documented a mean age of 50.8 years; and Laurinavicius et al. [18] reported that 47.71% of participants were younger than 55 years and 52.29% were older than 55 years.

In our study, infrared thermography demonstrated a specificity and PPV of 100%, while sensitivity was moderate at 75% and the NPV was 62.5%. The overall diagnostic accuracy was 82.35%, with an AUC of 0.875. These results are comparable to a 2016 study reporting a sensitivity of 81.6% and specificity of 57.8% [19], confirming the high specificity of thermography despite its relatively lower sensitivity.

Our findings showed complete exclusion of benign lesions, with no false-positive cases. These findings highlight the potential role of infrared thermography as a reliable adjunctive diagnostic modality, particularly in contexts where reducing false-positive diagnoses is essential. Recent advances in artificial intelligence (AI)-assisted thermography have further improved diagnostic performance. In a cohort of 459 women, the AI-driven Thermalytix system demonstrated a sensitivity of 95.24% and a specificity of 88.6%. At the same time, deep learning models such as ResNet152 combined with support vector machines (SVMs) have achieved AUC values above 99% [20]. Although conventional infrared thermography in our study demonstrated encouraging performance, it remains inferior to these AI-enhanced models, underscoring the potential advantages of AI integration.

In the literature, machine-learning-based rotational thermography protocols have demonstrated diagnostic accuracies of approximately 93% [21], whereas compressed thermal imaging prototypes have reported classification accuracies up to 97.6% [22]. Rassiwala et al. [23] achieved exceptionally high performance, with a sensitivity of 97.6%, a specificity of 99.17%, excellent predictive values, and an almost perfect NPV of 99.89%. Similarly, Bansal et al. [24] reported a sensitivity of 95.24% and a specificity of 88.58%, with a strong NPV of 99.74% and an AUC of 0.9316, although the PPV was relatively low at 28.57%. In contrast, Nair et al. [25] observed moderate diagnostic accuracy, with a sensitivity of 88.24% and a specificity of 70.52%. In comparison, Alikhassi et al. [16] demonstrated sensitivity of 85.7%, specificity of 78.5%, PPV of 15.8%, NPV of 99.1%, overall diagnostic accuracy of 78.8%, and an AUC of 0.821.

Compared with mammography, our findings reaffirm prior evidence indicating the superior specificity of mammography over infrared thermography [23]. In the present study, mammography achieved a sensitivity of 95.83%, confirming its strong diagnostic ability for breast cancer detection. However, specificity was limited to 50.00%, reflecting a higher incidence of false-positive results. The PPV was also 95.83%, while the NPV was 50.00%, with an overall diagnostic accuracy of 92.31%. Notably, the AUC value of 0.067 appears unexpectedly low and may represent a statistical or data processing anomaly. Other published studies have reported more balanced mammographic performance. Nair et al. [25] documented high sensitivity (96.25%) and specificity (96.70%) with excellent predictive values, while Prasad et al. [17] reported comparable sensitivity (95.38%). Our findings confirm the excellent sensitivity and PPV of mammography; however, the reduced specificity and NPV suggest a tendency toward overdiagnosis of benign lesions. These results reinforce the importance of multimodal imaging approaches to optimize diagnostic accuracy in the evaluation of breast cancer.

Limitations

This study has several limitations that should be considered when interpreting its findings. First, the sample size was relatively small (N = 34), which may have reduced the statistical power and limited the generalizability of the results. Second, being conducted at a single tertiary care center, the study may not fully capture population-level variability in patient demographics, disease presentation, and healthcare access. Third, the diagnostic performance of infrared thermography was influenced by environmental factors and operator expertise, which could have introduced variability in image acquisition and interpretation. Fourth, thermography was evaluated using conventional methods without integration of AI, potentially limiting its diagnostic capability compared with more advanced approaches. Finally, the study population was restricted to women aged 20-60 years with palpable breast lumps, thereby excluding asymptomatic individuals and older women who represent a critical target group for breast cancer screening.

## Conclusions

Infrared thermography demonstrated high specificity and a substantial PPV in this exploratory study, indicating potential utility as an adjunctive diagnostic tool in the evaluation of breast lesions. However, given its modest sensitivity, limited evidence regarding reproducibility and operator variability, and the absence of large-scale validation, thermography cannot replace mammography as a primary screening or diagnostic modality. Mammography continues to demonstrate superior overall diagnostic accuracy and remains the gold standard for breast cancer imaging. The present findings support the use of thermography only as a supplementary technique, not for population-based screening. Further large-scale, blinded, and stratified studies are required to confirm these observations and better define the clinical role of thermography.
